# Population Genetics, Genetic Structure, and Inbreeding of *Commiphora gileadensis* (L.) C. Chr Inferred from SSR Markers in Some Mountainous Sites of Makkah Province

**DOI:** 10.3390/plants12132506

**Published:** 2023-06-30

**Authors:** Hassan Mansour, Khalid H. Alamer, Zaki M. Al-Hasawi

**Affiliations:** 1 Biological Sciences Department, Faculty of Science and Arts, King Abdulaziz University, Rabigh 21911, Saudi Arabia; kalamer@kau.edu.sa (K.H.A.); zalhasawy@kau.edu.sa (Z.M.A.-H.); 2 Department of Botany and Microbiology, Faculty of Science, Suez Canal University, Ismailia 41522, Egypt; 3 Department of Biological Sciences, Faculty of Science, King Abdulaziz University, Jeddah 21589, Saudi Arabia

**Keywords:** *Commiphora gileadensis*, conservation, populations, genetic diversity, Makkah

## Abstract

*Commiphora gileadensis* (L.) C. Chr is a perennial plant existing mainly in the southern and western mountains of the Arabian Peninsula. In the Makkah province, the remaining populations are threatened by many factors such as overcutting, overgrazing, and urban developments. These dangers are expected to be aggravated by the progression of aridification factors arising from climate change. To overcome the decline in remaining populations of this valuable species, a timely evaluation of the population’s genetic variables and genetic structure is vital for the conservation of existing *C. gileadensis* populations. In this study, we used 61 SSR primers to achieve this objective. Only 50 loci showed polymorphisms, which led to further analysis of the population genetics for 600 genotypes that were collected from 50 populations of *C. gileadensis* found in 10 different sites in the Makkah region: Gebel Al Muliesaa, Wadi Albathna, Wadi Houra, Wadi Albaidaa, Wadi Elebiedia, Gebel Kniethl, Wadi Sayaa, Wadi Elbarasa, Wadi Alfawara, and Wadi Alkharar. The results showed an obvious decrease in genetic diversity variables in all studied populations. The range of *PPL* was between 8 and 40; additionally, the low *H*_T_ value of 0.804 and the high value of inbreeding, *F*_is_ = 0.238, reflected a severe lack of heterozygotes. High levels of F_ST_ and G_ST_ and low gene flow indicate considerable segregation among the *C. gileadensis* populations, which creates a barrier to gene migration. Our data suggest the need for conservation planning for *C. gileadensis* in order to avoid the species’ forthcoming extinction. Efforts should be largely oriented around managing water consumption, prohibiting overcutting and overgrazing, and establishing appropriate seed banks.

## 1. Introduction

Many members of the genus *Commiphora* are listed as endangered due to the overcollection of their populations for utilisation in medicinal and economical purposes (Burseraceae [Myrrh family]) [1]. *Commiphora gileadensis* (L.) C. Chr is considered one of the most economically and medicinally valuable trees grown in the Northern Hemisphere. Its distribution is mainly centred in the Red Sea region in the southern and western mountains of Saudi Arabia and other mountainous habitats in neighbouring countries within the Southern Arabian Peninsula and East Africa [2,3]. The well-known name of this tree in Saudi Arabia is “Besham” or Balsam, and its economic value is largely due to its use in the perfume industry [4,5] as well as its many medicinal applications, e.g., as a remedy for respiratory system diseases [6]. 

*Commiphora gileadensis* is found in few remaining populations, and it is mainly associated with the bottom of rocky mountains in the south and western regions of Saudi Arabia where its distribution is centred in Makkah province (Figure 3). The species was originally estimated to have between 1800 and 3000 populations that declined to the current estimate of only 60 populations (personal observation). The apparent reduction in the population numbers and population size of *C. gileadensis* could be attributed to progressive climate change conditions in the region [7] which, exacerbated by anthropogenic impacts [8,9], are anticipated to lead to a greater decline in the sizes of existing *C. gileadensis* populations and other associated plant taxa in arid habitats such as the Makkah region.

*Commiphora gileadensis* plants could be affected by a considerable decline in genetic diversity as a consequence of genetic drift, which is a key reason for the apparent low fitness and thus severe inability of many populations to adapt to ambient environmental confrontations [10,11,12]. Therefore, elucidating the population genetic variables and genetic structures of the remaining plant populations of *C. gileadensis* is crucial to conserve and restore this valuable plant species [13] and may support conservation plans for other plant taxa [14].

One of the main limiting factors for plant species with low population sizes is their potential for outcrossing and performing successful seed setting, especially under the stress of arid conditions. For *C. gileadensis*, self-incompatibility represents an extra stress that endangers its existence. This species has a floral structure like other members of Burseraceae and is recognizable by small, actinomorphic, and slightly odoriferous flowers; these features are considered to promote obligate outcrossing [15]. This reproductive system can severely impact population genetics corresponding to the survival of plant populations in arid habitats and can cause the loss of polymorphic genes, genetic drift, as well as progressive inbreeding [16,17].

As a result of the ambient climate change conditions connected with human over-utilisation in the Makkah province, the existing plant taxa—including remaining populations of *C. gileadensis*—are prone to the risk of extinction because of the ongoing decline in population size and potential loss of genetic diversity. Genetic analysis is pivotal for detecting genetic variation [18] using SSR (simple sequence repeats). Loci are considered to be of great value for measuring the gene diversity and genetic structures in collapsing plant populations of *C. gileadensis* due to their high potential to detect repeat regions with variable sequences in the target genome. These loci are well characterized as co-dominant molecular markers [19,20,21,22,23]. SSR markers were successfully applied to assess population genetics and genetic structure in other rare plant species [24,25,26].

Our research aims to elucidate the genetic diversity and genetic structure patterns of *C. gileadensis* populations under the xeric conditions of the Makkah province; by applying microsatellite loci, we can extract the required data for proposing mandatory conservation plans that are crucial to prevent the imminent threat of extinction for *C. gileadensis* and other associated plant species grown in this region.

## 2. Results

A total of 50 loci showed polymorphisms. The percentage of polymorphic loci (Table 1) was at its maximum value (40) in the Walb 5 and Walbd5 populations in Wadi Albathna and Wadi Albaidaa, respectively, whereas the minimum percentage of polymorphic loci (8) was detected in the Welbi1 population in Wadi Elebiedia. High selfing was indicated by our results for *C. gileadensis*, as the average inbreeding coefficient (F_is_) was 0.238, verifying an obvious deficit of heterozygotes (Table 1).

The mean number of alleles per locus (*N_a_*) varied between 1.48 (Walb 5 population) and 1.08 (Welbi1 population), resulting in the mean number of effective alleles per locus (N_e_) and the Shannon index (I). The highest number of private alleles was 0.087 and was calculated in the Gmul 3 population, while no private alleles were detected in Walb 1, Walb 4, Walbd5, Welbi2, Welbi3, Welbi5, Welbr2, Welbr3, Welbr5, Walk 2, and Walk 3 populations. Expected heterozygosity (H_e_) ranged from 1.281, 0.217, and 0.138, respectively, in the Gmul 3 and Walb 5 populations to 1.037, 0.039, and 0.025, respectively, in the Welbi1 and Walbd2 populations (Table 1). The average total heterozygosity (H_T_) for all loci and populations was equal to 0.804.

**Figure 1:**
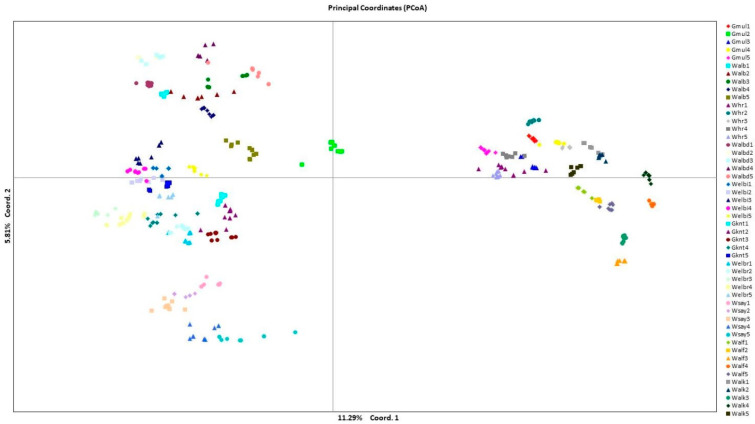
Principal coordinate analysis to categorize *C. gileadensis* populations based on pairwise genetic distance among 600 individual genotypes belonging to 50 studied populations with coloured and polygon codes for each population acronym.

The PCoA results (Figure 1) indicated that five out of seven principal components were significant (eigenvalue > 1) and considered as 99.9 of the sum variation. The main five significant components were N_a_, N_e_, I, the number of private alleles, and H_o_.

The analysis categorized the studied populations of *C. gileadensis* into four groups. The upper-right group comprised individuals who belonged to populations of Gebel Al Muliesaa, Wadi Alkharar, and Wadi Houra; the upper-left group contained individuals in populations from Wadi Albathna, Wadi Elebiedia, and Wadi Albaidaa; the lower-left group contained populations from Gebel Kniethl, Wadi Elbarasa, and Wadi Sayaa; and the lower-right group contained Wadi Alfawara populations.

**Figure 2:**
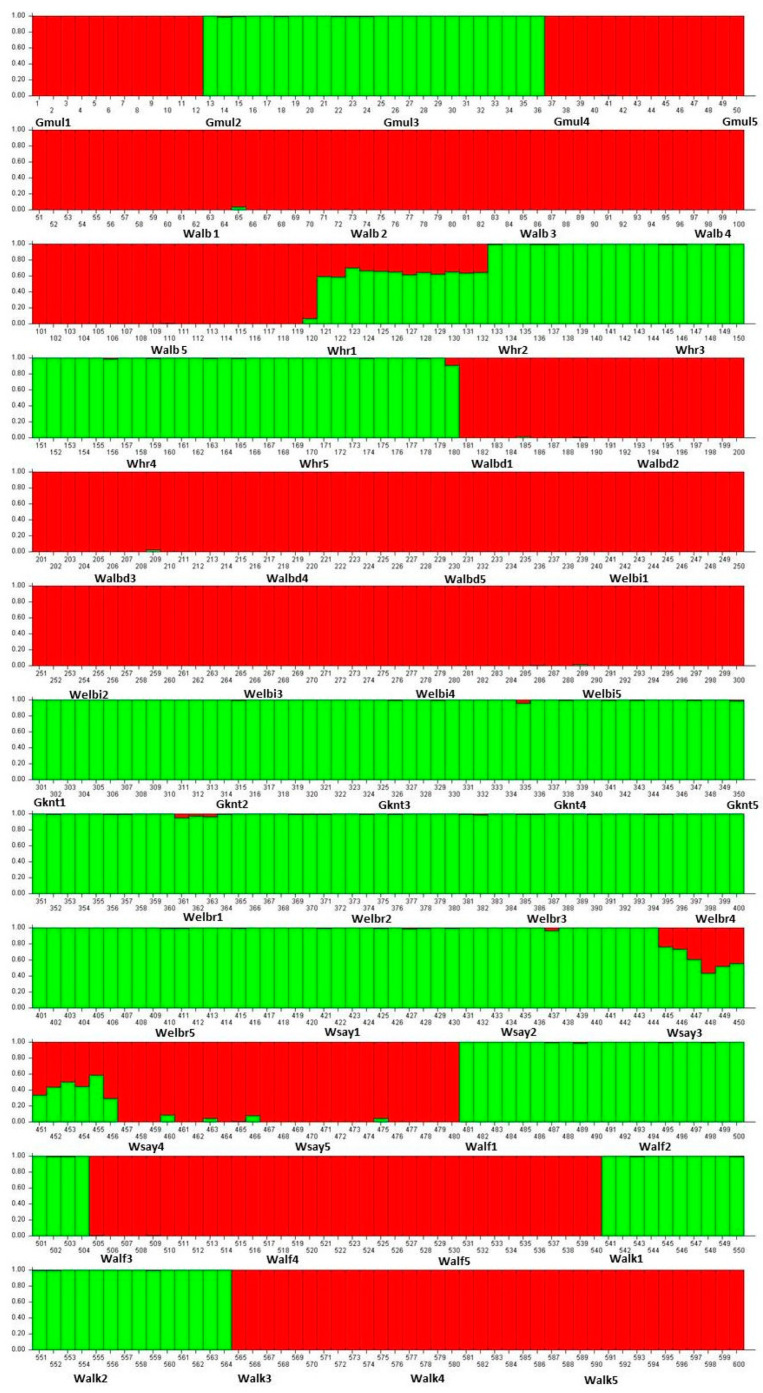
Population structure of 600 genotypes of *C. gileadensis* belonging to 50 populations that were grouped into two subgroups based on 50 SSR markers (K = 2); the acronyms of each population were written next to the genotypes where they belonged. The green colour referred for the first subgroup and the red colour referred to the second subgroup.

Evanno’s method [27] indicated that K = 2 was optimal among the 50 populations of *C. gileadensis* (Figure 2).

The AMOVA showed substantial genetic differentiation among the studied *C. gileadensis* populations where F_ST =_ 0.896 and was higher with *R*_ST_ = 0.980. The maximum genetic differentiation was observed between different populations (98, *p* = 0.001), while the minimum value (1, *p* = 0.010) was measured among individuals in the same population. The gene flow of Nm = 0.024 is a low value for the gene migration among population per generation. G-statistic measurements showed a higher value of F_ST_ = 0.913 and G_st_ value = 0.908 compared to the F_ST_ resulting from AMOVA, which confirmed high genetic differentiation among different populations.

## 3. Discussion

In this study, all the genetic diversity variables of *C. gileadensis* showed a modest to extreme decline in measurements among all the enduring populations analysed and was in agreement with other studies on closely related plant species of Burseraceae. This indicates an extensive decline in gene diversity and meets the corresponding severe environmental circumstances [28,29,30,31,32,33,34] confirmed by the comparison of the high genetic differentiation F_ST_ values, which measured for *C. gileadensis* (F_ST_ = 0.896) with other rare plant species in similar plant habitats in South Sinai, and they revealed considerable values of genetic differentiation, e.g., *Primula boveana* (F_ST_ = 0.737) [35] and *Cotoneaster orbicularis* (F_ST_ = 0.634) [32].

The main reason behind the lack of genetic diversity in the existing populations may be their small population size. A leading factor underlying this decline could be that *C. gileadensis* populations are subjected to high genetic drift and inbreeding, which exacerbate the problem of decline in polymorphic alleles as an imminent result of encountering environmental conditions [36,37,38].

The distribution outline of genetic diversity locations among the studied populations revealed a considerable range of variability, with relatively modest polymorphisms measured in the populations of Gebel Al Muliesaa, Wadi Alkharar, and Wadi Houra, which could be attributed to the relative abundance of water reserves in these regions. Wadi Albathna and Wadi Albaidaa are located at the foot of the Bany Ayoub Mountain region, where water reserves are more abundant due to frequent floods caused by rain in this location. Wadi Elbarasa has low-altitude valleys characterized by water aggregations that allow for the growth of a few plant populations. The slight gorges in Gebel Kniethl (750–1000 m a.s.l) facilitate the growth of plant populations [39], revealing the same connection between genetic diversity maintenance in rare plant populations and water availability in desert habitats.

The PCoA determined the association between the genetic diversity in *C. gileadensis* and the abundance of water reserves. The PCoA subdivided populations with relatively high polymorphisms, showing sites with water abundance on the right and left upper parts of the PCoA axis: Gebel Al Muliesaa, Wadi Alkharar, and Wadi Houra were in the upper-right group and populations from Wadi Albathna, Wadi Elebiedia, and Wadi Albaidaa were in the upper-left group. The STRUCTURE analysis results for the existence of only two subpopulations for the studied 500 genotypes of *C. gileadensis* and the notable values of genetic differentiation among populations, calculated using AMOVA and confirmed with G-statistics, indicated considerable isolation among the population sites. The main reason for this high isolation could be the increasing activities of human inhabitants, which include overcutting for cosmetic and medicinal purposes. This can also include excessive overgrazing by camels and sheep herds owned by local tribes in the area or other tribes inhabiting the southern region of Saudi Arabia with drier climatic conditions [40] that become extremely hazardous during spring. Moreover, water reserves are at risk of high depletion due to the recent increasing human populations associated with growing industries and petroleum refinery companies in the region [7,41].

Larger decreases in the population size of *C. gileadensis* and further isolation are anticipated with increasing temperatures and water deficiency conditions, as indicated by the fluctuations of rain frequency in these sites [8,9]. Moreover, the constant influence of increasing temperatures could pose a greater risk to the reproductive capabilities of *C. gileadensis* flowers—as well as have a negative impact on pollination potential—and is thus expected to increase selfing [33,42], as revealed by the excessive low values of the measured inbreeding coefficient (F_is_).

*C. gileadensis* is characterized by drupe-type fruit with one seed that is considered relatively heavy for wind dispersal (sizes range from 3.5 to 4.8 cm), which supports our computed low level of gene flow among *C. gileadensis* populations. For this reason, the extensive anthropogenic and climatic causes of isolation have promoted the elevation of the genetic differentiation value among the remaining populations of *C. gileadensis*. This phenomenon was clearly outlined in the PCoA. The values of the calculated gene flow decreased considerably from the values required for preventing an increase in genetic drift [43]. The concurrent influences of genetic drift and gene flow could aggravate the future drop in gene diversity among the remaining populations of *C. gileadensis*.

## 4. Materials and Methods

### 4.1. Plant Materials

**Figure 3:**
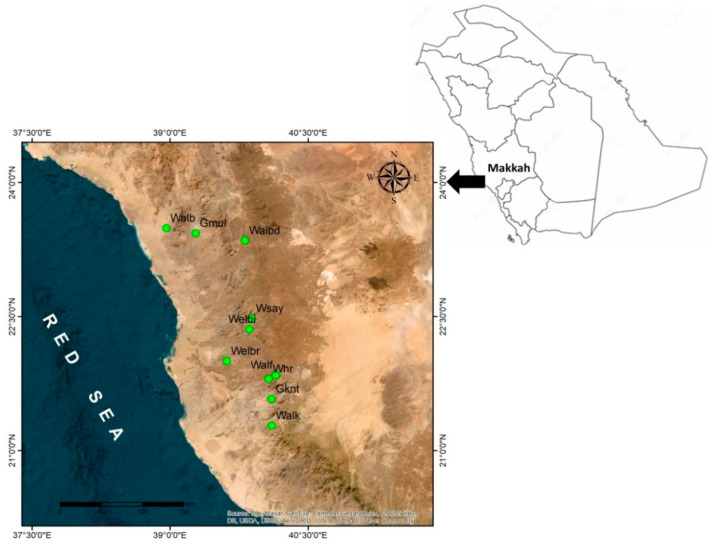
The studied sites of Commiphora gileadensis in the Makkah province, Kingdom of Saudi Arabia (Gmul: Gebel Al Muliesaa; Walb: Wadi Albathna; Whr: Wadi Houra; Walbd: Wadi Albaidaa; Welbi: Wadi Elebiedia; Gknt: Gebel Kniethl; Welbr: Wadi Elbarasa; Wsay: Wadi Sayaa; Walf: Wadi Alfawara; Walk: Wadi Alkharar).

The sampling of *C. gileadensis* in the mountains and plains of the Makkah province included fifty populations from ten different sites (Table 2, Figure 3). From every site, five populations were selected for sampling. All studied populations were located at the bottom of the rocky mountains between the Alabwaa village and the Makkah metropolitan area. The highest number of individuals was found in a population located in Wadi Albaidaa at the bottom of the Ayoub mountains east of Abwaa (Figure 2). The lowest number of individuals (16) was the Whr1 population in the Wadi Houra site, which was the result of extensive human activities in the area—such as overcutting—and overgrazing by sheep and cattle herds over the whole site.

Twelve individuals were sampled from each of the studied populations. Two to three leaflets were preserved directly in liquid nitrogen and then placed at −20 °C in a freezer for further DNA isolation.

### 4.2. Genomic DNA Extraction and PCR Tests

Isolation of DNA from the preserved leaflet samples for 600 plant individuals was performed using a DNeasy Plant Mini Kit (Qiagen, Germantown, MD, USA). Fifty loci revealing polymorphisms were recognized using sixty-one formerly published primers for other species belonging to the Burseraceae family [28,29,30,31]. The polymorphic primer tests were performed for all sampled individuals (Table S1). A master mix for PCR trials was prepared according to procedures outlined in [34]. PCR tests were carried out using a C1000 Thermal Cycler (BioRad, Hercules, CA, USA). The PCR reaction conditions were as follows: initial denaturation at 95 °C for 5 min; followed by 45 cycles at 94 °C for 35 s for denaturation; 55 °C for 40 s for annealing; and 72 °C for 5 min for final extension.

The products of the PCR reactions were sequenced using a 3130xl Genetic Analyzer (Applied Biosystems, Foster City, CA, USA) with LIZ500 as a size standard. The sequences of amplified fragments were determined using GeneMapper 4.0 (Applied Biosystems, Foster City, CA, USA), and the lengths of the amplified fragments ranged from 112 to 300 bp in accordance with [44].

### 4.3. Population Genetic Analysis

The variables of genetic diversity, genetic structure, inbreeding, and G-statistics were calculated using GenAlEx 6.5 [45], measuring the genetic differentiation among the populations with RST for microsatellite loci [46].

The genetic structure for 50 populations of *C. gileadensis* was conducted using the Bayesian clustering method in STRUCTURE version 2.3.4 [47], with the admixture model implemented and K values (number of potential clusters) ranging from one to ten. The burn-in period and Markov Chain Monte Carlo (MCMC) conducted 100,000 iterations [27]. The optimal K value was determined using the method of Evanno et al. [27] as implemented in STRUCTURE Harvester [48].

AMOVA was applied with 999 permutations to assess genetic differentiation among populations [49,50]. Gene flow (*Nm*) was calculated via the private allele method [51]. The analysis of heterozygosity (*H*_o_), the expected heterozygosity (*H*_e_) under Hardy–Weinberg equilibrium, and Wright’s fixation index (*F* = 1 − *H*_o_/*H*_e_) were tested for each locus in each population to test deviations from the Hardy–Weinberg equilibrium and thereby determine inbreeding in existing populations of *C. gileadensis*. Principal coordinate analysis (PCoA) was performed using GenAlEx 6.5 [45] based on pairwise genetic distance data among 600 individual genotypes belonging to 50 studied populations.

## 5. Conclusions and Recommendations

Our current research represents the first assessment of distribution patterns of population genetic variables and genetic structures among and within the remaining populations of *C. gileadensis* in habitats of the Makkah region; it was carried out in order to contribute to the conservation management and protection for *C. gileadensis* as an economically and medicinally valuable plant species. This species is confronted by the danger of forthcoming extinction due to an extreme loss of gene polymorphism associated with excessive interpopulation genetic differentiation and severe inbreeding.

The conservation plan of *C. gileadensis* should be based on long-term and progressive actions and should be oriented mainly to prevent the continuous degradation of its populations and its habitats. Many efforts could be considered in this area. Firstly, the results of the present study suggest that we should establish enclosures of a wire-fence type to protect populations that are subjected to severe low genetic polymorphisms [52], as indicated in our study for Wadi Alkharar, Wadi Albaidaa, Wadi Alfawara, and Gebel Al Muliesaa. Such enclosures are crucial to prevent the dangers of overgrazing from camels and sheep in these locations; they should be monitored regularly to observe vegetation parameters and detect any further alterations in the protected populations. Secondly, management plans for water resources should be proposed to promote water utilisation, enhancing the reuse of wastewater and the effective usage and storage of rainwater and underground water. These plans should be incorporated into undergraduate learning programs in educational institutes, in addition to increasing public awareness for managing water consumption among inhabitants of the Makkah region.

Thirdly, the apparent decline in genetic diversity and the considerable genetic differentiation in these populations could be reclaimed via performing a test of interpopulation crosses among the populations with the highest genetic differentiation values (F_ST_) and higher selfing, potentially increasing the population fitness [53]. Many factors should be considered during the design of the interpopulation crosses to alleviate the consequences of outbreeding depression, such as the genetic distance between the concerned populations, their latitude, and the genetic diversity magnitude, e.g., crosses between extremely low genetic diversity populations resulted in the outbreeding value in F1 [54]. Moreover, a test bank for the collected seeds from the studied remaining populations can be established [53]. The collected seeds should be planted in greenhouses as nurseries, and seedlings with strong vegetative characteristics could be planted into populations with the lowest genetic diversity resembling that of the populations from which the parent seeds were collected; this would reduce potential consequences, including further inbreeding and an extreme decline of gene flow. Parts of the collected viable seeds should be preserved using suitable procedures for seed maintenance in well-equipped test banks; these test banks would be of high value for future *C. gileadensis* conservation efforts in its habitats. The recommended measures for the conservation of *C. gileadensis* could be applicable to the other rare species in the genus *Commiphora* which grow in similar arid habitats.

## Figures and Tables

**Table 1:** The measurements of population genetic variables of *C. gileadensis* populations across studied sites in Makkah province.

Population	N_a_	N_e_	I	No. of Private Alleles	Ho	He	P	F_is_
Gmul 1	1.200	1.103	0.092	0.066	0.073	0.058	18.00	−0.221
Gmul 2	1.320	1.144	0.139	0.057	0.083	0.085	26.00	0.136
Gmul 3	1.440	1.281	0.209	0.087	0.143	0.131	32.00	−0.073
Gmul 4	1.380	1.166	0.145	0.066	0.078	0.084	26.00	0.163
Gmul 5	1.340	1.172	0.155	0.052	0.083	0.097	30.00	0.064
Walb 1	1.140	1.062	0.066	0.000	0.040	0.042	14.00	−0.031
Walb 2	1.300	1.163	0.151	0.028	0.042	0.098	28.00	0.583
Walb 3	1.240	1.144	0.122	0.020	0.047	0.079	20.00	0.317
Walb 4	1.220	1.080	0.085	0.000	0.013	0.052	22.00	0.691
Walb 5	1.480	1.229	0.217	0.000	0.112	0.138	40.00	0.152
Whr 1	1.340	1.180	0.162	0.057	0.133	0.104	30.00	−0.123
Whr 2	1.340	1.161	0.140	0.064	0.057	0.084	26.00	0.335
Whr 3	1.180	1.061	0.067	0.059	0.050	0.041	18.00	0.009
Whr 4	1.280	1.124	0.117	0.039	0.065	0.071	22.00	0.162
Whr 5	1.160	1.059	0.068	0.052	0.035	0.042	16.00	0.234
Walbd1	1.120	1.056	0.054	0.028	0.043	0.034	10.00	−0.147
Walbd2	1.100	1.037	0.040	0.000	0.018	0.025	10.00	0.092
Walbd3	1.240	1.133	0.120	0.000	0.037	0.079	22.00	0.438
Walbd4	1.120	1.037	0.047	0.000	0.007	0.028	12.00	0.796
Walbd5	1.400	1.129	0.148	0.028	0.031	0.089	40.00	0.767
Welbi1	1.080	1.039	0.039	0.028	0.010	0.025	8.00	0.429
Welbi2	1.240	1.079	0.091	0.000	0.032	0.053	20.00	0.305
Welbi3	1.260	1.152	0.139	0.000	0.037	0.092	26.00	0.528
Welbi4	1.260	1.112	0.107	0.020	0.038	0.066	20.00	0.393
Welbi5	1.240	1.118	0.115	0.000	0.087	0.072	20.00	−0.204
Gknt 1	1.420	1.256	0.189	0.070	0.142	0.113	30.00	−0.245
Gknt 2	1.420	1.187	0.162	0.044	0.072	0.093	30.00	0.330
Gknt 3	1.340	1.227	0.160	0.062	0.055	0.098	20.00	0.415
Gknt 4	1.420	1.241	0.195	0.020	0.077	0.121	30.00	0.347
Gknt 5	1.360	1.208	0.155	0.070	0.080	0.091	24.00	0.160
Welbr1	1.240	1.141	0.108	0.034	0.057	0.065	16.00	0.032
Welbr2	1.180	1.079	0.080	0.000	0.030	0.049	14.00	0.428
Welbr3	1.240	1.149	0.129	0.000	0.000	0.087	24.00	1.000
Welbr4	1.280	1.166	0.147	0.044	0.052	0.097	26.00	0.417
Welbr5	1.200	1.110	0.105	0.000	0.025	0.069	20.00	0.619
Wsay1	1.160	1.077	0.073	0.048	0.050	0.047	16.00	0.007
Wsay2	1.120	1.071	0.062	0.000	0.020	0.041	12.00	0.667
Wsay3	1.260	1.121	0.118	0.028	0.055	0.075	24.00	0.440
Wsay4	1.160	1.090	0.083	0.020	0.033	0.055	16.00	0.357
Wsay5	1.180	1.119	0.102	0.020	0.042	0.069	18.00	0.434
Walf 1	1.240	1.135	0.113	0.028	0.088	0.070	18.00	−0.233
Walf 2	1.100	1.051	0.048	0.000	0.037	0.031	10.00	0.089
Walf 3	1.240	1.124	0.113	0.044	0.077	0.070	20.00	−0.099
Walf 4	1.140	1.084	0.065	0.020	0.027	0.042	10.00	0.409
Walf 5	1.220	1.130	0.117	0.039	0.095	0.078	22.00	−0.139
Walk 1	1.240	1.120	0.106	0.020	0.073	0.065	18.00	−0.139
Walk 2	1.140	1.062	0.056	0.000	0.022	0.034	10.00	0.412
Walk 3	1.180	1.101	0.095	0.000	0.072	0.062	18.00	−0.158
Walk 4	1.140	1.052	0.050	0.028	0.015	0.031	10.00	0.416
Walk 5	1.220	1.136	0.114	0.069	0.070	0.073	18.00	−0.024
Overall mean	1.245	1.125	0.112	0.029	0.055	0.070	20.60	0.238

**Table 2:** Information of the studied sites and populations of *Commiphora gileadensis* in Makkah province (acronym of 3–4 letters refers to population name, and the number refers to different populations within same site).

Population Acronym	Population Site	Longitude (E)	Latitude (N)	Altitude (m.)	Total No. of Individuals
Gmul 1	Gebel Al Muliesaa	39°16′43″	23°26′03″	216	22
Gmul 2					30
Gmul 3					19
Gmul 4					20
Gmul 5					15
Walb 1	Wadi Albathna	38°57′32″	23°29′22″	371	19
Walb 2					29
Walb 3					38
Walb 4					33
Walb 5					30
Whr 1	Wadi Houra	40°04′18″	21°48′16″	612	16
Whr 2					26
Whr 3					30
Whr 4					35
Whr 5					22
Walbd1	Wadi Albaidaa	39°48′54″	23°21′07″	600	48
Walbd2					36
Walbd3					30
Walbd4					42
Walbd5					33
Welbi1	Wadi Elebiedia	39°51′43″	22°21′31″	551	44
Welbi2					40
Welbi3					33
Welbi4					49
Welbi5					31
Gknt 1	Gebel Kniethl	40°06′19″	21°34′45″	762	38
Gknt 2					31
Gknt 3					22
Gknt 4					37
Gknt 5					35
Welbr	Wadi Elbarasa	39°37′07″	22°00′09″	320	44
Welbr					22
Welbr					29
Welbr					26
Welbr					35
Wsay1	Wadi Sayaa	39°53′32″	22°28′42″	629	39
Wsay2					36
Wsay3					30
Wsay4					22
Wsay5					28
Walf 1	Wadi Alfawara	40°09′02″	21°50′42″	721	32
Walf 2					40
Walf 3					33
Walf 4					23
Walf 5					18
Walk 1	Wadi Alkharar	40°06′26″	21°16′48″	747	34
Walk 2					33
Walk 3					21
Walk 4					32
Walk 5					29

## Data Availability

Not applicable.

## Supplementary Materials

The following supporting information can be downloaded at: https://www.mdpi.com/article/10.3390/plants12132506/s1, Table S1: The tested primers status for *Commiphora gileadensis*.

## References

[1] Mathur M., Mathur P., Purohit H. (2023). Ecological niche modelling of a critically endangered species *Commiphora wightii* (Arn.) Bhandari using bioclimatic and non-bioclimatic variables. Ecol. Process.

[2] Miller A.G., Morris M. (1988). Plants of Dhofar, the Southern Region of Oman: Traditional, Economic and Medicinal Uses.

[3] Wood J.R.I. (1997). A Handbook of the Yemen Flora. With Color Illustrations by Hugo Haig-Thomas.

[4] Shen T., Li G.H., Wang X.N., Lou H.X. (2012). The genus Commiphora: A review of its traditional uses, phytochemistry and pharmacology. J. Ethnopharmacol..

[5] Mahr D. (2012). *Commiphora*: An Introduction to the Genus. Cactus Succul. J..

[6] González-Tejero M.R., Casares-Porcel M., Sánchez-Rojas C.P., Ramiro-Gutiérrez J.M., Molero-Mesa J., Pieroni A., Giusti M.E., Censorii E., De Pasquale C., Della A. (2008). Medicinal plants in the Mediterranean area: Synthesis of the results of the project Rubia. J. Ethnopharmacol..

[7] Tarawneh Q.Y., Chowdhury S. (2018). Trends of Climate Change in Saudi Arabia: Implications on Water Resources. Climate.

[8] Issar A.S., Zereini F., Hötzl H. (2008). The impact of global warming on the water resources of the Middle East: Past, present and future. Climate Changes and Water Resources in the Middle East and North Africa.

[9] Soultan A., Wikelski M., Safi K. (2019). Risk of biodiversity collapse under climate change in the Afro-Arabian region. Sci. Rep..

[10] Luijten S.H., Dierick A., Gerard J., Oostermeijer B., Raijmann L.E.J., Den Nijs H.C.M. (2000). Population size, genetic variation, and reproductive success in a rapidly declining, self-incompatible perennial (*Arnica montana*) in the Netherlands. Conserv. Biol..

[11] Hansson B., Westerberg L. (2002). On the correlation between heterozygosity and fitness in natural populations. Mol. Ecol..

[12] Bastiaan S., Hamish G.S. (2003). Effects of genetic drift and gene flow on the selective maintenance of genetic variation. Genetics.

[13] Shalabi L.F., Otaif F.S. (2022). *Commiphora* Jacq (Burseraceae) in Saudi Arabia, Botanical, Phytochemical and Ethnobotanical Notes. Ecologies.

[14] Hatmaker E.A., Staton M.E., Dattilo A.J., Hadziabdic D., Rinehart T.A., Schilling E.E., Trigiano R.N., Wadl P.A. (2018). Population Structure and Genetic Diversity within the endangered species *Pityopsis ruthii* (Asteraceae). Front. Plant Sci..

[15] Raju A.J.S., Lakshmi P.V., Ramana K.V., Chandra P.H. (2012). Entomophily, ornithophily and anemochory in the self-incompatible *Boswellia ovalifoliolata* Bal. & Henry (Burseraceae), an endemic and endangered medicinally important tree species. J. Threat. Taxa.

[16] Keller L.F., Waller D.M. (2002). Inbreeding effects in wild populations. Trends Ecol. Evol..

[17] Vilas C., San Miguel E., Amaro R., García C. (2005). Relative contribution of inbreeding depression and eroded adaptive diversity to extinction risk in small populations of shore campion. Conserv. Biol..

[18] Kadam U.S., Lossie A.C., Schulz B., Irudayaraj J. (2013). Gene expression analysis using conventional and imaging methods. DNA and RNA Nanobiotechnologies in Medicine: Diagnosis and Treatment of Diseases.

[19] Kim K.S., Sappington T.W., Kantartzi S. (2013). Microsatellite Data Analysis for Population Genetics. Microsatellites. Methods in Molecular Biology (Methods and Protocols).

[20] Upadhyay A., Kadam U.S., Chacko P., Karibasappa G.S. (2010). Microsatellite and RAPD analysis of grape (*Vitis* spp.) accessions and identification of duplicates/misnomers in germplasm collection. Indian J. Hortic..

[21] Upadhyay A., Kadam U.S., Chacko P.M., Aher L., Karibasappa G.S. (2010). Microsatellite analysis to differentiate clones of Thompson seedless grapevine. Indian J. Hortic..

[22] Hinge V.R., Shaikh I.M., Chavhan R.L., Deshmukh A.S., Shelake R.M., Ghuge S.A., Dethe A.M., Suprasanna P., Kadam U.S. (2022). Assessment of genetic diversity and volatile content of commercially grown banana (*Musa* spp.) cultivars. Sci. Rep..

[23] Chavhan R.L., Sable S., Narwade A.V., Hinge V.R., Kalbande B.B., Mukherjee A.K., Chakrabarty P.K., Kadam U.S. (2023). Multiplex molecular marker-assisted analysis of significant pathogens of cotton (*Gossypium* sp.). Biocatal. Agric. Biotechnol..

[24] Szczecińska M., Sramko G., Wołosz K., Sawicki J. (2016). Genetic Diversity and Population Structure of the Rare and Endangered Plant Species *Pulsatilla patens* (L.) Mill in East Central Europe. PLoS ONE.

[25] Yu Y.L., Wang H.C., Yu Z.X., Schinnerl J., Tang R., Geng Y.P., Chen G. (2021). Genetic diversity and structure of the endemic and endangered species *Aristolochia delavayi* growing along the Jinsha River. Plant Divers.

[26] Chen L., Pan T., Qian H., Zhang M., Yang G., Wang X. (2021). Genetic Diversity and Population Structure Revealed by SSR Markers on Endemic Species *Osmanthus serrulatus* Rehder from Southwestern Sichuan Basin, China. Forests.

[27] Evanno G., Regnaut S., Goudet J. (2005). Detecting the number of clusters of individuals using the software structure: A simulation study. Mol. Ecol..

[28] Misiewicz T.M., Barbosa C.E., Fine P.V. (2012). Microsatellite primers for an Amazonian lowland tropical tree, *Protium subserratum* (Burseraceae). Am. J. Bot..

[29] Maradani B.S., Gudasalamani R., Setty S., Chandrasekaran R. (2018). Development of microsatellite markers for the resin-yielding, non-timber forest product species *Boswellia serrata* (Burseraceae). Appl. Plant Sci..

[30] Koffi K.G., Heuertz M., Jans R., Hardy O.J., Vendramin G.G., Duminil J. (2012). Characterization of new microsatellite loci isolated from *Santiria trimera* (Burseraceae). Am. J. Bot..

[31] Rimlinger A., Marie L., Avana M.L., Bouka G.U., Zekraoui L., Mariac C., Carrière S.M., Duminil J. (2020). New microsatellite markers for *Dacryodes edulis* (Burseraceae), an indigenous fruit tree species from Central Africa. Mol. Biol. Rep..

[32] Mansour H., Sliwinska E. (2017). Genetic Diversity and Inbreeding Level of Cotoneaster orbicularis Schltdl. in The Sinai Mountains Revealed by Microsatellite Markers and Flow Cytometry. Egypt. J. Bot..

[33] Mansour H., Alsamadany H., Al-Hasawi Z.M. (2020). Genetic diversity and genetic structure of *Salvadora persica* L., rare plant species in Rabigh province, Saudi Arabia: Implications for conservation. J. Taibah Univ. Sci..

[34] Mansour H., Alsamadany H., Al-Hasawi Z.M. (2022). Molecular Assessment of Genetic Diversity and Genetic Structure of *Rhanterium epapposum* Oliv. in Scarce Populations in Some Regions of Western Saudi Arabia. Plants.

[35] Jimenez A., Mansour H., Keller B., Conti E. (2014). Low genetic diversity and a high level of inbreeding in the Sinai primrose (*Primula boveana*), a species on the brink of extinction. Plant Syst. Evol..

[36] Blomqvist D., Pauliny A., Larsson M., Flodin L.A. (2010). Trapped in the extinction vortex? Strong genetic effects in a declining vertebrate population. BMC Evol. Biol..

[37] Jacquemyn H., Roldán-Ruiz I., Honnay O. (2010). Evidence for demographic bottlenecks and limited gene flow leading to low genetic diversity in a rare thistle. Conserv. Genet..

[38] Smyser T.J., Duchamp J.E., Johnson S.A., Larkin J.L., Rhodes O.E. (2012). Consequences of metapopulation collapse: Comparison of genetic attributes between two Allegheny woodrat metapopulations. Conserv. Genet..

[39] Al-Gharaibeh M.M., Hamasha H.R., Rosche C., Lachmuth S., Wesche K., Hensen I. (2017). Environmental gradients shape the genetic structure of two medicinal *Salvia* species in Jordan. Plant Biol..

[40] Al-Rowaily S.L., El-Bana M.I., Al-Bakre D.A., Assaeed A.M., Hegazy A.K., Ali M.B. (2015). Effects of open grazing and livestock exclusion on floristic composition and diversity in natural ecosystem of Western Saudi Arabia. Saudi. J. Biol. Sci..

[41] Harter T., Davis H., Mathews M., Meyer R. (2022). Shallow ground water quality on dairy farms with irrigated forage crops. J. Contam. Hydrol..

[42] Root T.L., Price J.T., Hall K.R., Schneider S.H., Rosenzweig C., Pounds J.A. (2003). Fingerprints of global warming on wild animals and plants. Nature.

[43] Spieth P.T. (1974). Gene flow and genetic differentiation. Genetics.

[44] Arif I.A., Khan H.A., Shobrak M., Al Homaidan A.A., Al Sadoon M., Al Farhan A.H., Bahkali A.H. (2010). Interpretation of electrophoretograms of seven microsatellite loci to determine the genetic diversity of the Arabian Oryx. Genet. Mol. Res..

[45] Peakall R., Smouse P.E. (2012). GenAlEx v.6.5: Genetic analysis in Excel. Population genetic software for teaching and research. Bioinformatics.

[46] Slatkin M. (1995). A measure of population subdivision based on microsatellite allele frequencies. Genetics.

[47] Pritchard J., Stephens M., Rosenberg N., Donnelly P. (2000). Association mapping in structured populations. Am. J. Hum. Genet..

[48] Earl E.A., Von Holdt B.M. (2012). Structure Harvester: A website and program for visualizing STRUCTURE output and implementing the Evanno method. Conserv. Genet. Res..

[49] Excoffier L., Smouse P.E., Quattro J.M. (1992). Analysis of molecular variance inferred from metric distances among DNA haplotypes: Application to human mitochondrial DNA restriction data. Genetics.

[50] Michalakis Y., Excoffier L. (1996). A generic estimation of population subdivision using distances between alleles with special reference for microsatellite loci. Genetics.

[51] Barton N.H., Slatkin M. (1986). A Quasi-equilibrium theory of the distribution of rare alleles in a subdivided population. Heredity.

[52] Koyama A., Uchida K., Ozeki M., Iwasaki T., Nakahama N., Suka T. (2021). Conservation of endangered and rare plants requires strategies additional to deer-proof fencing for conservation of sub-alpine plant diversity. Appl. Veg. Sci..

[53] Oakley C.G., Lundemo S., Ågren J., Schemske D.W. (2019). Heterosis is common and inbreeding depression absent in natural populations of Arabidopsis thaliana. J. Evol. Biol..

[54] Holsinger K.E., Gottlieb L.D., Falk D.A., Holsinger K.E. (1991). Conservation of rare and endangered plants: Principles and prospects. Genetics and Conservation of Rare Plants.

