# Supplementary material for: Population Genetics, Genetic Structure, and Inbreeding of Commiphora gileadensis (L.) C. Chr Inferred from SSR Markers in Some Mountainous Sites of Makkah Province
Source: Plants (Basel). 2023 Jun 30;12(13):2506. doi: 10.3390/plants12132506 (PMC10346734; doi:10.3390/plants12132506)
Supplement: Supplementary file 1 [file plants-12-02506-s001.zip › plants-2335065-supplementary.pdf]

Table S1. The tested primers status for *Commiphora gileadensis*.

Marker		Ta(°C)	SSR motif	Size of PCR products	References
Prot02	F: ATAAACCCTCTTACGGTGAG<br>R: GGGATTTGTTGACTTTGAAC	60/45	$(ACAT)_6$	170–230	[19]
Prot08	F: TATGTCCCACAATGATCCTC<br>R: TTTATAGGAGCGCTCTGATC	60/45	$(ACAT)_7$	186–242	
Prot13	F: TGATTTCTTGTCCCAAAGAG<br>R: AGCCACATACCGATAAACTC	60/45	$(AAAG)_7$	309–311	
Prot22	F: TAACCCTTGACAAGCATTTC<br>R: AAATTACGGCTTCAGAATTG	60/45	$(AGAT)_9$	326–360	
Prot28	F: CGCAGTTTCAGAAATATCAG<br>R: GCATGATTCGATGTTATAGG	60/45	$(ACAT)_8$	238–261	
Prot29	F: TGAACTGACCTTTGCATGAC<br>R: AAGAGGGTGGTCTGAACTG	60/45	$(AAAG)_{15}$	128–172	
Prot67	F: TCATGCTGTAATTCCCTGTC<br>R: GAGAAGAGCAAAGATTCGATAG	60/45	$(ACAT)_7$	191–239	
Prot70	F: CCATTATTAAGCATGCAAAC<br>R: CAATGGCCTGTTCATATAAAG	60/45	$(AAG)_9$	111–255	
Prot71	F: CCATCCTCAGCTCTTACTTTC<br>R: GATCGGTCACAGATTCAATG	60/45	$(AAG)_8$	398–422	
Prot78	F: CACACCAGGAAAGACTCAAG<br>R: TTGGAAGGAGGATTTATAGG	60/45	$(AAG)_8$	139–148	
Prot83	F: CGTCTGGATGGAAGATAAAG<br>R: TCCTCGTTCTCCACTACAAC	60/45	$(AAC)_{10}$	161–189	
Prot97	F: ATTCCGATTAAACCTCATTC<br>R: GGGTATGAGCTTGAATTAGG	60/45	$(AG)_{13}$	166–196	
Prot99	F: ATGCTATGATAATCGGTTCC<br>R: GAAATGGTTGCACTTCACTC	60/45	$(AG)_{12}$	149–209	
Prot100	F: ATCTCTCGTTCCAACTCAAC<br>R: CGTCGAAGTACTCACCACTC	60/45	$(AC)_{10}$	178–190	
Prot101	F: CATTTAGGGACCACGTTTAC<br>R: ATTGTTCCAGGATCTAGGTG	60/45	$(AG)_{11}$	288–292	
Prot102	F: GTCGACCAAATAATGTCACC<br>R: ATGGACACACAGGACCTATC	60/45	$(AG)_{15}$	370–388	
Prot104	F: TAACCGCAATATCAACTCTC<br>R: ACACCACGACTAAAGACTGG	60/45	$(AC)_{11}$	281–299	
BS6	F: CTACGTATTGATGAGGCGGC<br>R: GAGATCGATGGAATTGCTGGT	60	$(GA)_{14}$	178–239	[20]
BS8	F: CGCCTCAGCTCACCAGTAAT<br>R: CTGCCAGAGTATGAGAAGCAA	52	$(CAAAA)_4…(CAA)_4$	180–210	
BS10	F: ACACGGGTCTGAACTCCAAC<br>R: GGATGATTCATTCCTGGAAAA	60	$(AAG)_{20}$	198-222	
BS11	F: AACAACCAACCCATCTCACTC<br>R: GGTCGGTTGAGGATGGAATA	54	(TC)7	182–216	
BS12	F: ATCGGGTGATTCTGCTATCG<br>R: GCAGAATCGCTACTCGATGA	58	$(AG)_9$	170-199	
BS13	F: TTCCTTGGACATAGCCAAATCT<br>R: GTAGACCTAGCATCTTCAGCTG	58	$(AG)_{11}$	278–303	
BS14	F: CCGGCGCAACTTCAATAATA<br>R: ATCAGCAAGTCCGTCTGTCC	58	$(AAC)_4$	192–222	
BS16	F: CGCTTTCTATTTTCCTTTTTGG<br>R: GCTAACGATTGACAACTGCTG	50	$(CT)_{15}$	112–144	

BS18	F: CAACGAGAGGAGGCAGTGAT<br>R: TTAAGGCCTGGCTAGCAGAA	58	$(TC)_{30}$	188-202	
BS19	F: GGATCCAGCCGCAGTATATC<br>R: TCGACAGCTCAAGGAATGTG	56	$(TG)_{32}$	212-233	
BS21	F: CAGCCTTCCTCAATCGGATA<br>R: AAGTCGGTCACCTCATTGGA	52	$(TGT)_{12}$	211-233	
BS23	F: CACGATGACGTGATTCTGCT<br>R: CAAGCTTGCACACAGGAAAA	52	$(CA)_{9}$	191–266	
BS25	F: TCAAGCCGTTGTAGTTGGTG<br>R: TGGAAGCACAGAAAGAAGCA	52	$(TC)_{15}$	200–244	
BS28	F: CCAGCATTTTTCCTTTCTTTTT<br>R: TTGCTCACGAAATCCTTCCT	56	$(AAG)_{13}$	154–181	
BS29	F: CACGATGACGTGATTCTGCT<br>R: CTTACACCATCTCCCTCTGC	52	$(AG)_{9}$	178–238	
BS32	F: CTGCCAGGCCTTAAACAAAA<br>R: GCAGTGGATGGGGTAGAATC	60	$(TG)_{17}$	240–277	
Santri04	F: AGCAGTGTTGGTTCAGGAGAA<br>R: AGCTTCTGCTCCACCTGATG	63.7	$(TG)_{9}$	133–148	[21]
Santri07	F: CAGCATGAGGTTGATTGGAC<br>R: CAACCTTGCCTACTGTTTCG	62.4	$(CA)_{8}$	188–233	
Santri10	F: Q4-AAGAGAAGCATGGATGACGG<br>R: TGAGGTGGTCCAGACTTTCC	63.7	$(AG)_{11}$	202–247	
Santri15	F: TTCATCTGGGTCAGCCGTAT<br>R: CTCAACAAATCCCGCTTCTC	62.3	$(GAA)_{8}$	267–288	
Santri16	F: Q3-GAATCCATCTTTCCCTTAGCAG<br>R: CCATTGAGTTGCCTCCAAAT	61.8	$(TTC)_{8}$	266–322	
Santri20	F: GCCCTTCTAAGAAATCACCCA<br>R: CATTTACACGAGCATTCACATT	61.7	$(CA)_{8}$	370–412	
Santri26	F: Q3-CTTGATTCCTTCCTTGACGG<br>R: CACGAGGGATGAGAGCAACT	64.9	$(TC)_{10}$	152–168	
DaE-14	F: GGCGGCCATGTTTGTGAAAT<br>R: TACGTAACGGCGATTCAGTTT	53	$(AT)_{8}$	122–139	[22]
DaE-24	F: AGGCGTAGAAATCCCATCCA<br>R: ATCTCCAGGAACGGTTGACT	53	$(AT)_{8}$	150–163	
DaE-34	F: TGTGTTTCTTCCGCCTCACA<br>R: ATGAACTGCAGCCTCAAACG	53	$(AT)_{7}$	169–190	
DaE-5[c]	F: AGGATGTTGGTCTGTTGGTGG<br>R: TGGCAGAGGAGAGGATAAGCA	53	$(AT)_{8}$	101–152	
DaE-7	F: AAGAAGGCAAGGCAACACC<br>R: CAGAGCACACTGTACTGGCA	53	$(AG)_{8}$	110–163	
DaE-28	F: CAGAGGTGGGAATCTTAA GCCA<br>R: AACACTGCTTCAGCTTTCAGA	53	$(AG)_{15}$	151–172	
DaE-39	F: ACCCATTTGAGCTCATTTGACA<br>R: TGCCACTGGGAGTAGAAGAGT	53	$(AT)_{16}$	169–216	
DaE-13	F: ATGGCCATTCTCACACCTGG<br>R: ACAGTTGATTTCCATTCCACCC	53	$(AT)_{7}$	138–169	
DaE-19	F: GCAAGATTGTGGTGTTGCTGT<br>R: GTGACTCGGAGAAAGCGGTT	53	$(AAC)_{9}$	118–157	
DaE-27	F: TTCAATATTGTGGCTGGTCCCA<br>R: TCAGCTCTCAGACAAAGGCA	53	$(AG)_{15}$	150–213	
DaE-36	F: TCATTGAGCCACAAGGGAAC<br>R: ACAGAGGAGTGTGATCGAAGA	53	$(AT)_{10}$	160–223	
DaE-41	F: TGGTTACTTCAAGCACAGAGAC<br>R: CTGCAATATCGATGCCTCTTCT	53	$(AT)_{11}$	199–252	
DaE-16	F: CTACTCGTGACTGCTCTTGC<br>R: GGAATGACCGTGTTGTTTGGG	53	$(AG)_{20}$	115–166	

DaE-20	F: AGATCCCTCTGAATCCCTGATT<br>R: TCATGTCTTCCCTCTTTAGGCT	53	$(AT)_9$	130–191
DaE-23	F: CAGCGAACCCATGTCAAACG<br>R: TCCTCAAAGCTACCCTATCCCA	53	(AG)13	133–248
